# Complete genome sequence and characterisation of a novel flexivirus infecting the necrotrophic conifer pathogen *Diplodia sapinea*

**DOI:** 10.1007/s00705-025-06313-6

**Published:** 2025-05-16

**Authors:** Suvi Sutela, Miloš Trifković, Eeva Terhonen

**Affiliations:** 1 Natural Resources Institute (Luke), Natural Resources, Forest Health and Biodiversity, Helsinki, Finland; 2 Department of Forest Protection and Wildlife Management, Faculty of Forestry and Wood Technology, Mendel University in Brno, Brno, Czech Republic

## Abstract

Using total RNA sequencing, we detected a novel RNA virus infecting a Finnish isolate of the fungal conifer pathogen *Diplodia sapinea*, and its genome sequence was verified by Sanger sequencing. This virus, which we have named "Diplodia sapinea flexi-like virus 1" (DsFLV1), has a 7,494-nucleotide-long positive-sense single-stranded RNA genome, excluding the poly(A) tail. The largest open reading frame (ORF1) encodes a replication-associated polyprotein (RP) that contains conserved domains characteristic of members of the order *Tymovirales*, including viral methyltransferase (Mtr), viral helicase (Hel), and RNA-dependent RNA polymerase (RdRP) domains. Four smaller ORFs were also identified. ORF2 potentially encodes a capsid protein, while the other three ORFs, initiated by alternative start codons, encode putative proteins with unknown functions. Phylogenetic analysis placed DsFLV1 in a distinct clade within the family *Deltaflexiviridae*, close to Erysiphe necator-associated flexivirus 2. This is the first report of a positive-sense single-stranded RNA virus infecting *D. sapinea*.

**Supplementary Information:**

The online version contains supplementary material available at 10.1007/s00705-025-06313-6.

*Diplodia sapinea* (Fr.) Fuckel (syn. *Sphaeropsis sapinea* (Fr.) Dyko & B. Sutton) is an ascomycetous conifer pathogen found worldwide that predominantly infects pines. This fungus has a dynamic lifestyle, switching between endophytic (latent infection), pathogenic, and saprotrophic stages [1, 2]. Mycoviruses infect fungal species across all major taxa [3], and most of them cause cryptic infections. However, some mycoviruses can cause hypovirulence in their fungal host, and the mycovirus Cryphonectria hypovirus 1 has been used successfully for biological control of chestnut blight [4, 5]. *D. sapinea* was one of the first pine pathogens to be examined for mycoviral infection, and two different totiviruses have been found in this species [6]. While further studies have found dsRNA elements in this fungus [7, 8], RNA viruses have been reported only in other members of the genus *Diplodia* [9–11].

With one exception [12], all of the members of the family *Deltaflexiviridae*, like other members of the order *Tymovirales*, possess a (+) single-stranded (ss) RNA genome ranging in length from 5.9 to 9.0 kb that is 5′-m7G-capped and 3′-polyadenylated. The largest, 5′-proximal, open reading frame (ORF) encodes a replication-associated polyprotein (RP) [13]. Until recently, deltaflexiviruses were considered to be capsidless [14]. The family currently has only one genus, *Deltaflexivirus*, with four species recognised by ICTV [15–18]. Other putative deltaflexiviruses infecting phytopathogenic fungi have been described [19–21]. Here, we report a newly discovered mycovirus infecting the Finnish *D. sapinea* strain 138. Its genome organisation (Fig. 1A) and phylogenetic placement show that it is closely related to members of the family *Deltaflexiviridae*, and we have therefore named it "Diplodia sapinea flexi-like virus 1" (DsFLV1).

**Fig. 1:**
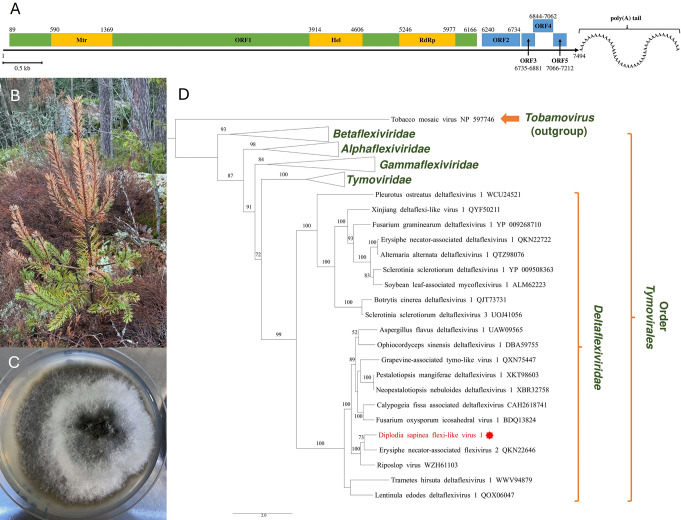
(**A**) Schematic representation of the genome of Diplodia sapinea flexi-like virus 1 (DsFLV1). The RP ORF is shown in green, and the smaller putative ORFs are shown in blue. Yellow rectangular boxes indicate conserved domains (Mtr, viral methyltransferase; Hel, viral RNA helicase; RdRP, catalytic core domain of RNA-dependent RNA polymerase conserved in + ssRNA viruses and closely related viruses). (**B**) Symptomatic Scots pine shoot with Diplodia tip blight from which D. sapinea was isolated. (**C**) D. sapinea strain 138 after cultivation at 20°C for 14 days. (**D**) ML (RAxML) phylogenetic tree based on amino acid sequences of the DsFLV1 RP (in red with an asterisk) and those of other members of the order *Tymovirales* (Supplementary Table S3). Bootstrap values ≥ 50% are shown at the nodes. Branch lengths represent the number of aa substitutions per site. Scale bar, 2.0 substitutions per site per branch. The tree is rooted at the midpoint

*Diplodia sapinea* strain 138 was isolated from a symptomatic shoot (Fig. 1B) of a Scots pine seedling from South Finland [22]. Briefly, DNA from a pure fungal culture (Fig. 1C) originating from surface-sterilized twigs was isolated using PrepMan Ultra Sample Preparation Reagent (Applied Biosystems). The species identification was confirmed by amplification using the species-specific primers DiSapi-F and Diplo-R (Supplementary Table S1) and Sanger sequencing (Macrogen Europe) of the ITS1-5.8S-ITS2 region of the rDNA (Supplementary Table S1). Two-week-old hyphae were collected from cellophane-covered 2% potato dextrose agar, frozen at -80°C, and freeze-dried. Total RNA was extracted using a QIAGEN RNeasy Plant Mini Kit, treated with DNase I (Thermo Fisher Scientific), cleaned using Zymo RNA Clean & Concentrator (Zymo Research), and sent to CeGaT GmbH (Tübingen, Germany) for library preparation using a TruSeq Stranded mRNA Kit (Illumina) and sequencing. For amplification of the 5’ and 3’ untranslated regions (UTRs) the DNase-I-treated total RNA was ligated with a T4 adapter [23, 24] using T4 RNA ligase (Thermo Fisher Scientific) and purified using Zymo RNA Clean & Concentrator following the protocol for the purification of large RNAs. The adapter-ligated RNA was converted to cDNA using Maxima H Minus Reverse Transcriptase (Thermo Fisher Scientific), and non-ligated DNase-I-treated total RNA was converted to cDNA using RevertAid Reverse Transcriptase (Thermo Fisher Scientific). The amplification was performed using Phusion Plus Green PCR master mix (Thermo Fisher Scientific) using virus-specific primer pairs or a T4-adapter-specific primer with a 5’ or 3’ UTR-specific primer (Supplementary Table S1). The PCR products were purified using NucleoSpin Gel and PCR Clean-up (Macherey-Nagel), sequenced by the Sanger method at Macrogen Europe, and assembled using Geneious Prime 2025.0.3 (Biomatters). Each position in the viral sequence was determined twice, using two independent cDNAs. Bioinformatics, sequence analysis, and construction of the phylogenetic tree were performed as described previously [25].

*De novo* assembly of 35,838,692 raw reads (BioProject PRJNA1215369; BioSample SAMN46401780) using Trinity-v2.15.1 produced three long virus-like contigs corresponding to sequences from members of the family *Deltaflexiviridae*. With confirmed 5′ and 3′ UTRs, 7,331,769 raw reads (20% of the total) were mapped to the complete DsFLV1 sequence of 7,494 nt (GenBank ID PQ876383) without the poly(A) tail (Fig. 1, Supplementary Fig. S1). Sanger sequencing of the whole viral genome revealed the presence of seven polymorphic sites (Supplementary Table S2, Supplementary Fig. S1) as well as a 35-nt-long sequence not found in all amplicons, possibly representing a subgenomic RNA (Supplementary Fig. S1).

The largest ORF (ORF1) of DsFLV1 is 6,078 nt in length (nt 89-6166) and encodes a putative RP of 2025 amino acids (aa) with a deduced molecular weight (*Mr*) of 226.1 kDa. NCBI Conserved Domain Database (CDD) searches revealed three conserved domains: viral RNA methyltransferase (Mtr; nt 590–1369; E-value, 3.41e-14), viral RNA helicase (Hel; nt 3914–4606; E-value, 6.73e-10), and RNA-dependent RNA polymerase (RdRP; nt 5246–5977; E-value, 6.32e-35) (Supplementary Fig. S2), which are characteristics of members of the family *Deltaflexiviridae*. An Mtr domain close to the predicted N-terminal region of the RP suggests that the genomic RNA may be capped. NCBI BLASTp analysis showed that the deduced aa sequence of ORF1 shared a high degree of sequence similarity with the RPs of other deltaflexiviruses. Short, incomplete sequences from the database were excluded from the search results. The closest related virus (identity, 60.76%; E-value, 0.0; coverage, 97%) was Erysiphe necator associated flexivirus 2 (EnaFV2; GenBank ID QKN22646). The putative RP of DsFLV1 also showed similarity (identity, 58.88%; E value, 0.0; coverage, 93%) to the RP of Riposlop virus (GenBank ID WZH61103). Three of the four shorter ORFs, ORFs 3–5 were predicted to use an alternative start codon, CUG, for initiation of translation. ORF2 (nt 6240–6734), located 73 nt downstream of ORF1, encodes a putative 164-aa protein with a predicted *Mr* of 17.2 kDa, with closest similarity to a hypothetical protein of grapevine wood associated deltaflexivirus 2 (GenBank ID XLE34830; identity, 29.67%; E-value, 1.7e-3; coverage, 55%) according to a BLASTp search. A search for proteins with similar structure and function using HHblits (database UniRef30_2023_02) (https://toolkit.tuebingen.mpg.de/tools/hhblits) [26] yielded a high-probability hit (97.38%; E-value, 6.8e-6) with the capsid protein (CP) of soybean leaf-associated mycoflexivirus 1 [17] (UniRef entry A0A0S1WF58). A BLAST search (https://www.uniprot.org/blast) of the UniProtKB database showed similarity of the ORF2 aa sequence (identity, 36%; E-value, 3.6e-9) to the CP of Sclerotinia sclerotiorum deltaflexivirus 1 [16] (UniProtKB entry A0A125R920). Given that other deltaflexiviruses and related viruses have been shown to possess icosahedral nucleocapsids [14], we hypothesise that ORF2 encodes the putative capsid protein of DsFLV1 (see Supplementary Fig. S3 for an aa alignment). ORF3 (nt 6735–6814) and ORF4 (nt 6844–7062) encode short putative proteins of 48 aa (5.5 kDa) and 72 aa (8.4 kDa), respectively. The last CUG-initiated ORF (ORF5) is located four nt downstream from ORF4 (nt 7066–7212; 48 aa; 5.3 kDa). No conserved domains or significant similarities based on BLASTp were detected in the sequences of the proteins encoded by ORFs 3–5.

A maximum-likelihood phylogenetic analysis of RP aa sequences of DsFLV1 and related viruses of the order *Tymovirales* showed that the viruses in the family *Deltaflexiviridae* formed a distinct cluster, separate from the other four families in the order (Fig. 1D). DsFLV1 was placed in a distinct monophyletic cluster supported by a high bootstrap value, with its closest relative being EnaFV2. This supports the proposal of Wu *et al*. [14] to establish a new viral family within the order *Tymovirales* to host the first “flexivirus” shown to be encapsidated. When completely characterised, these viruses, alongside DsFLV1, may represent additional species in the new family. To our knowledge, DsFLV1 is the first + ssRNA virus reported to infect *D. sapinea*.

## Electronic Supplementary Material

Below is the link to the electronic supplementary material

Supplementary Material 1

## Data Availability

The RNAseq raw reads can be found under BioSample no. SAMN46401780 (BioProject PRJNA1215369), and the DsFLV1 genome sequence is available under GenBank ID PQ876383.
