# Supplementary material for: Complete genome sequence and characterisation of a novel flexivirus infecting the necrotrophic conifer pathogen Diplodia sapinea
Source: Arch Virol. 2025 May 16;170(6):131. doi: 10.1007/s00705-025-06313-6 (PMC12084271; doi:10.1007/s00705-025-06313-6)
Supplement: Supplementary file 1 — Supplementary Material 1 [file 705_2025_6313_MOESM1_ESM.pdf]

Supplementary Table S1. Primers utilized in the current study

Purpose	Forward primer	Sequence of forward primer	Reverse primer	Sequence of reverse primer
Host determination	ITS1-F[a]	CTTGGTCATTTAGAGGAAGTAA	ITS4[b]	TCCTCCGCTTATTGATATGC
Host determination	DiSapi-F[c]	CCCTTATATATCAAACTATGCTTTGT	Diplo-R[c]	TTACATAGAGGATTGCCTTCG
Amplification of virus genome	Ds_flexi_q_L1	CAAGGCCTGCAATGCTCTCC	Ds_flexi_R1	CACTCGAACGTTACGCCTGC
Amplification of virus genome	Ds_flexi_L2	GAGAATGTTTGCGCCTCACA	Ds_flexi_R2	CGCAAGCAGAGTGAAGAACC
Amplification of virus genome	Ds_flexi_L2	GAGAATGTTTGCGCCTCACA	Ds_flexi_R2	CGCAAGCAGAGTGAAGAACC
Amplification of virus genome	Ds_flexi_L3	CCAAGCAGGCCATTGACTTT	Ds_flexi_R3	GCGCGTCTGATTGAGTTCAT
Amplification of virus genome	Ds_flexi_L4	TGCCTTCCCAGATTCATTCC	Ds_flexi_R4b	AAGGGAAGTGGGTAAGGACA
Sequencing primer for virus genome	Ds_flexi_1086F	CTATGTGTTCGCGGGTTCTG		
Sequencing primer for virus genome	Ds_flexi_3142F	CTGGTGCTCAATGTTACGCA		
Sequencing primer for virus genome	Ds_flexi_4843F	GCATACCAGAGCGTCTCCTC		
Sequencing primer for virus genome	Ds_flexi_6139L	CTTTTCGCCAATGCCCTTCC		
Sequencing primer for virus genome			Ds_flexi_1742R	ACGAAGGTGGAGGCAGTAAA
Sequencing primer for virus genome			Ds_flexi_3410R	GCGTGGATGGCATCTGAAAT
Sequencing primer for virus genome			Ds_flexi_4968R	ACTCCTTGTCGTGCCAATGG
Sequencing primer for virus genome			Ds_flexi_6810R	AACGGGAAAGCGACGAGAAT
Sequencing primer for virus genome			Ds_flexi_6318R	TCGGAGGGTCAATGAAAGGC
Determination of 5' UTR region	T4 primer[d]	AACCCGGGTCGAATGC	Ds_flexi_q_BegRev1	ACACAGGTGCATGGTCCAGA
Determination of 5' UTR region	T4 primer[d]	AACCCGGGTCGAATGC	Ds_flexi_BegRev2	GGACAAGTCACAATCGCGAA
Determination of 3' UTR region	Ds_flexi_EndFor1	TTGCCCTCCTCGTACCATTT	T4 primer[d]	AACCCGGGTCGAATGC
Determination of 3' UTR region	Ds_flexi_EndFor2	CGAGGAGGAGGAGGAAAGTG	T4 primer[d]	AACCCGGGTCGAATGC
Determination of 3' UTR region	Ds_flexi_EndF3	CATTACCATTCACCATAGCGCTCC	T4 primer[d]	AACCCGGGTCGAATGC
Determination of 3' UTR region	Ds_flexi_EndF4	CTTGGGGTGTGTTGCAGTGG	T4 primer[d]	AACCCGGGTCGAATGC

[a]White T, Bruns T, Lee S, Taylor J (1990) Amplification and direct sequencing of fungal ribosomal-RNA genes for phylogenetics. In: Innis MA, Gelfand DH, Sninsky JJ, White TJ (eds) PCR protocols: a guide to methods and applications. Academic Press, pp 315–322. https://doi.org/10.1016/b978-0-12-372180-8.50042-1.

[b]Gardes M, Bruns TD (1993) ITS primers with enhanced specificity for higher fungi and basidiomycetes: application to identification of mycorrhizae and rusts. Mol Ecol 2: 113–118. https://doi.org/10.1111/j.1365-294X.1993.tb00005.x.

[c]Adamson K, Laas M, Blumenstein K, Busskamp J, Langer GJ, Klavina D, Kaur A, Maaten T, Mullett MS, Müller MM, Ondrušková E, Padari A, Pilt E, Riit T, Solheim H, Soonvald L, Tedersoo L, Terhonen E, Drenkhan R (2021) Highly clonal structure and abundance of one haplotype characterise the Diplodia sapinea populations in Europe and Western Asia. J Fungi 7, article id 634. https://doi.org/10.3390/jof7080634.

[d]Modified after Lambden et al. (1992) by Tuomivirta and Hantula (2003):
Lambden, P.R., Cooke, S.J., Caul, E.O., Clarke, I.N., 1992. Cloning of noncultivatable human rotavirus by single primer amplification. J. Virol. 66, 1817–1822. Tuomivirta, T.T., Hantula, J., 2003. *Gremmeniella abietina* mitochondrial RNA virus S1 is phylogenetically related to the members of the genus *Mitovirus*∗. Arch Virol 148, 2429–2436.

Supplementary Table S2. Polymorphic sites of Diplodia sapinea flexi-like virus 1 (DsFLV1) based on sanger sequences and Find Variations/SNPs feature of Geneious Prime using 0.25 as the minimum variant frequency and 10^{-6} as the maximum variant P-value.

	Sangers	RNAseq data				
nt site	SNP	change	coverage	polymorphism type[a]	variant frequency	variant P-value
36	A/T	T -> A	356	transversion	83.40 %	0
252		C -> A	1537	transversion	77.30 %	0
486	C/T	C -> T	1676	transition	86.90 %	0
1892	G/A	G -> A	440	transition	67.00 %	0
2171	C/T	C -> T	499	transition	25.90 %	4.80E-304
23862	T/C	T -> C	385	transition	30.90 %	0
3447	C/A	C -> A	928	transversion	65.90 %	0
6280		CACT -> GTGG	10153 -> 17763	substitution[a]	67.4% -> 84.4%	0
6678		G -> A	302298	transition	99.20 %	0
6951		A -> G	430078	transition	93.50 %	0
7206	T/C	T -> C	1127840	transition	37.30 %	0

[a]All polymorphism types were SNP except for the substitution on site 6280

Supplementary Table S3. The viruses used in the maximum-likelihood tree.

Virus family	Virus name	GenBank ID
Alfaflexiviridae	Donkey orchid symptomles svirus	YP_008828152
	Sclerotinia sclerotiorum debilitation-associated RNA virus	YP_325662
	Indian citrus ringspot virus	NP_203553
	Allium virus X	YP_002647027
	Potato virus X	YP_002332929
	Lolium latent virus	YP_001718499
	Alfalfa virus S	QJD13457
	Garlic virus A	NP_569126
	Botrytis virus X	NP_932306
Betaflexiviridae	Banana mild mosaic virus	NP_112029
	Cherry green ring mottle virus	NP_047278
	Poplar mosaic virus	NP_958171
	Hop mosaic virus	YP_001798592
	Yam latent virus	YP_009134730
	Pepper virus A	YP_009357230
	Potato virus S	YP_277428
	Grapevine rupestris stem pitting-associated virus	NP_047281
	Apple chlorotic leaf spot virus	NP_040551
	Citrus leaf blotch virus	NP_624333
	Potato virus T	YP_002019748
	Grapevine virus A	NP_619662
	Apple stem grooving virus	NP_044335
Gammaflexiviridae	Botrytis virus F	NP_068549
	Sclerotinia sclerotiorum gammaflexivirus 1	QUE49151
	Apple virus D	QIC52853
	Leucocoprinus gammaflexivirus C	QED42996
	Monosporascus cannonballus	RYO87574
	Entoleuca gammaflexivirus 1	AVD68667
	Pistacia-associated flexivirus 1	QDO72745
	Dothistroma septosporum gammaflexivirus 1	WPV70693
Tymoviridae	Grapevine fleck virus	NP_542612
	Plantago mottle virus	YP_002308445
	Diascia yellow mottle virus	YP_002048673
	Tomato blistering mosaic virus	YP_008318042
	Eggplant mosaic virus	NP_040968

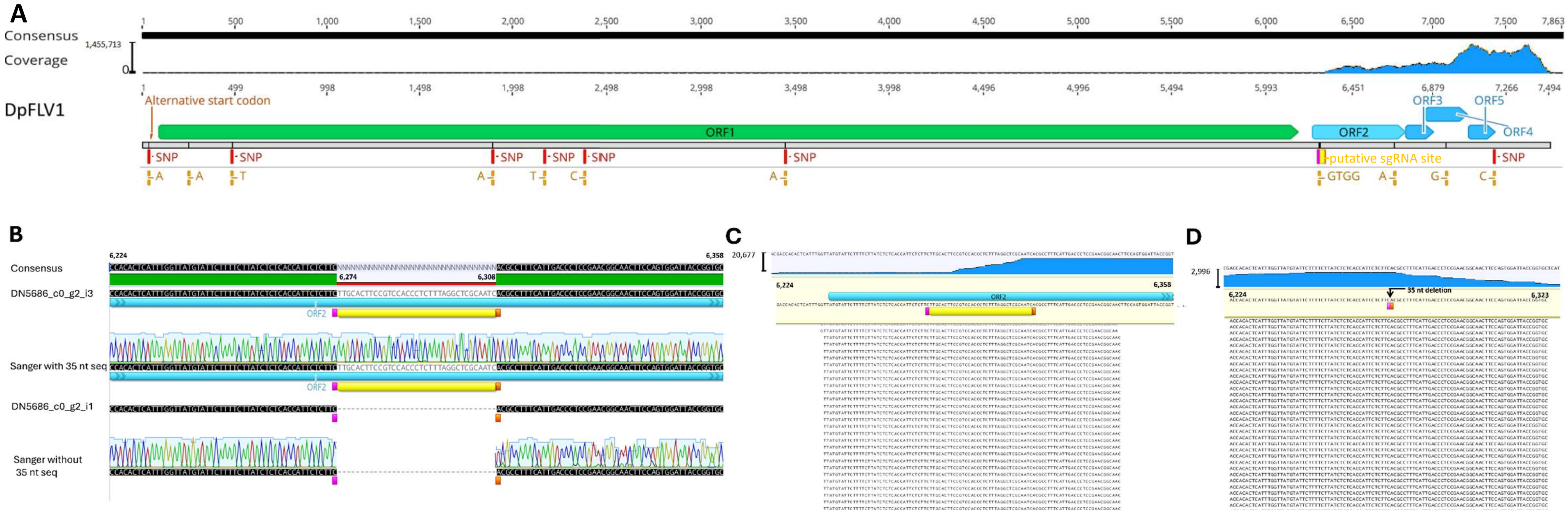

Supplementary Figure S1. A) Mapping of RNA-seq raw reads against the sanger-verified complete genome of Diplodia sapinea flexi-like virus 1 (DsFLV1) without poly(A) tail. Mapping was conducted with Geneious Prime 2025.0.3 (Biomatters) using Geneious RNA assembler with custom sensitivity (maximum mismatches per read 5%; no gaps allowed). The ORF1 of RP is marked with green, putative CP with light blue and smaller putative ORFs (3-5) starting with alternative codon CTG with blue. There was also alternative CTG start for ORF1 in position 47–49 marked with orange. Coverage (indicated with blue) of ORF1 (~300-8,200; average 1516) was considerably lower than the coverage of sequence of the putative ORFs 2–5 and the 3' UTR varying from 5,000 to ~1,400,000. The SNPs detected based on the sanger sequences are marked with red vertical lines. Orange lines represent the SNPs detected with the Find Variations/SNPs feature of Geneious Prime using 0.25 as the minimum variant frequency and 10^{-6} as the maximum variant P-value. The four SNPs GTGG were positioned to the putative site of subgenomic RNA (sgRNA; marked with yellow). **B)** Alignment of Trinity contigs (DN5686_c0_g2_i3 and DN5686_c0_g2_i1) with sanger sequences produced from DsFLV1 using the same primer pair. Both the Trinity contigs and sanger sequences have versions with 35 nt long sequence (marked with yellow; 5'-TTGCACTTCCGTCCACCCTCTTTAGGCTCGCAATC-3'; nts next to the sgRNA site marked with violet (5') and orange (3')) as well as sequences without the seq indicating presence of sgRNA. Based on the mapping the raw reads to sequences with **(C)** and without 35 nt seq **(D)** showed higher abundance of raw reads matching the longer sequence (21,735 raw reads; average coverage 9,356 reads) than to the shorter (3,954 raw reads; average coverage 1,617 reads) when conducted with Geneious RNA assembler with custom sensitivity (maximum mismatches per read 0%; no gaps allowed). Blue ORF denotes ORF2 encoding putative CP found present in DsFLV1 sequence when the 35 nt seq is included. Without the 35 nt seq, there is an alternative initiation (CTG) for the ORF2 at 6,337 nt. The figures originate from Geneious Prime.

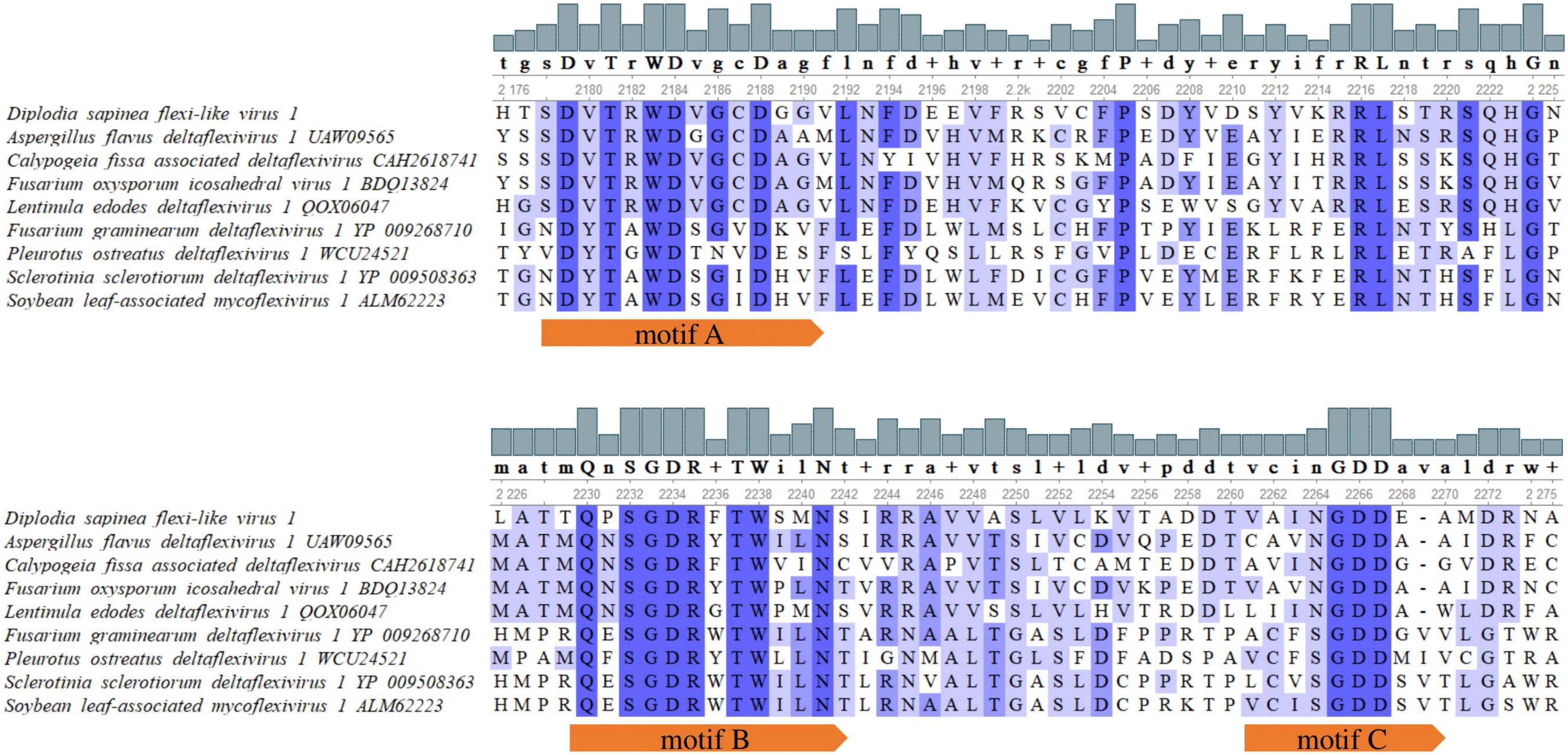

Supplementary Figure S2. Multiple alignment of amino acids sequences demonstrating conserved motifs A to C within the RdRp of DsFLV1, DsFLV1-related viruses and recognized virus species from the *Deltaflexiviridae* family. The alignment was done using MUSCLE[a] under Unipro UGENE 52.0[b]. The level of conservation is illustrated through bars placed above the alignment.

[a]Edgar, R.C., 2004. MUSCLE: a multiple sequence alignment method with reduced time and space complexity. BMC Bioinformatics 5, 113. https://doi.org/10.1186/14712105-5-113.

[b]Okonechnikov, K., Golosova, O., Fursov, M., Team, the U., 2012. Unipro UGENE: a unified bioinformatics toolkit. Bioinformatics 28, 1166–1167. https://doi.org/10.1093/bioinformatics/bts091.

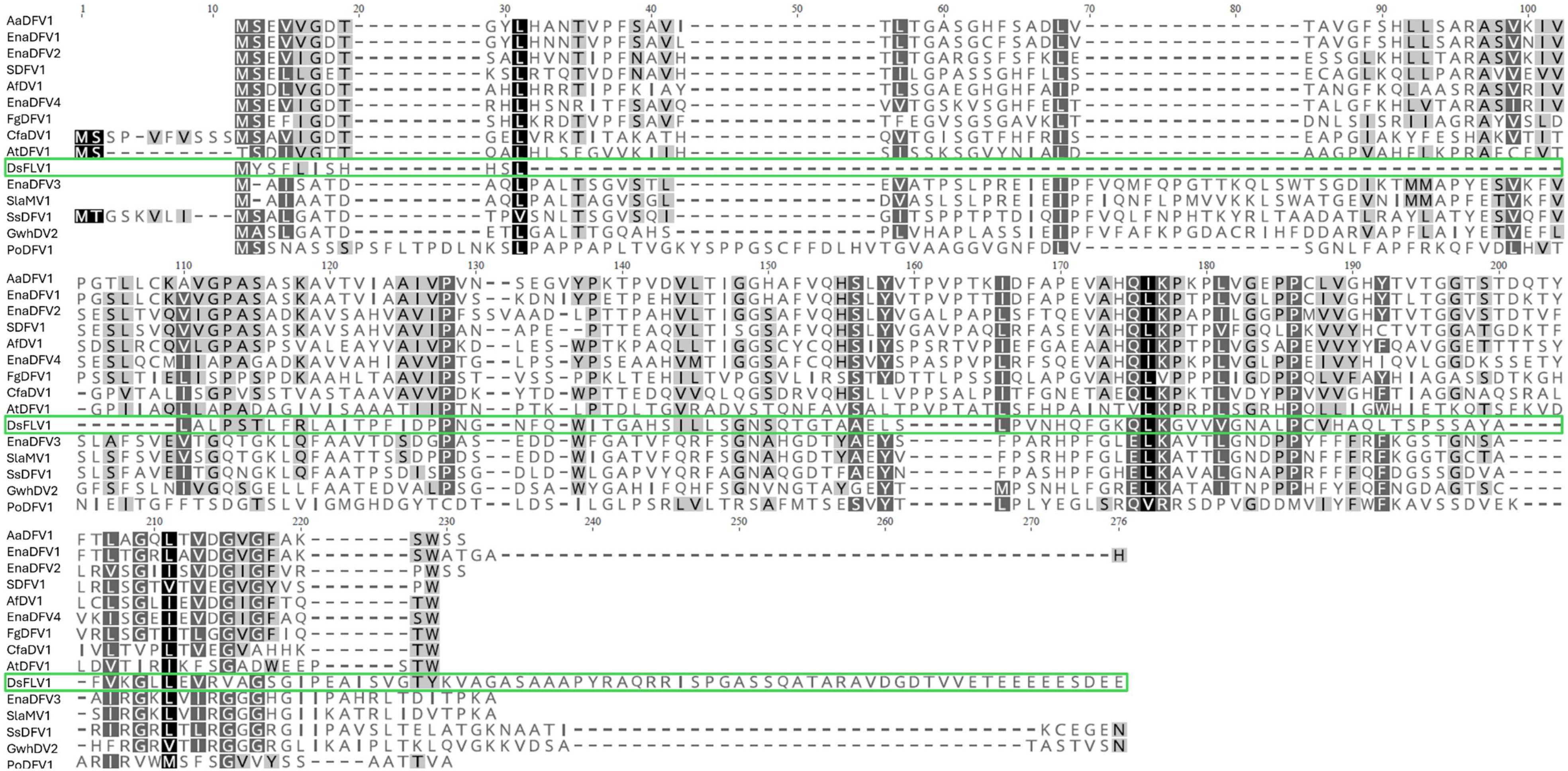

Supplementary Figure S3. Alignment (Mafft v7.490 Geneious Prime) of putative CP of Diplodia sapinea flexi-like virus (DsFLV1, highlighted with green) with CPs identified by Wu et al. (2024)[a]. Colors indicate similarity scored with Blosum62. Sequences: Alternaria alternata deltaflexivirus 1 (AaDFV1, QTZ98078), Aspergillus flavus deltaflexivirus (AfDFV1, translated from LC763238), Agrostis stolonifera deltaflexivirus 1 (AsDFV, QQG34630), Agave tequilana deltaflexivirus 1 (AtDFV1, QQG34635), Calypogeia fissa associated deltaflexivirus (CfaDFV, CAH2618743), Erysiphe necator associated deltaflexivirus 1 (EnaDFV1, QKN22723), Erysiphe necator associated deltaflexivirus 2 (EnaDFV2, QKN22648), Erysiphe necator associated deltaflexivirus 3 (EnaDFV3, translated from MN627465), Erysiphe necator associated deltaflexivirus 4 (EnaDFV4, QKN22696), Fusarium graminearum deltaflexivirus 1 (FgDFV1, YP_009268713), Pleurotus ostreatus deltaflexivirus 1 (PoDFV1, WCU24519), Sesame deltaflexivirus 1 (SDFV1, QQG34643), Soybean leaf-associated mycoflexivirus 1 (SlaMV1, YP_009508375), Sclerotinia sclerotiorum deltaflexivirus 1 (SsDFV1, YP_009508364).

[a]Wu C-F, Okada R, Neri U, et al (2024) Identification of a novel mycovirus belonging to the “flexivirus”-related family with icosahedral virion. Virus Evolution 10:veae093. https://doi.org/10.1093/ve/veae093dgar
